# Silibinin Inhibits HIV-1 Infection by Reducing Cellular Activation and Proliferation

**DOI:** 10.1371/journal.pone.0041832

**Published:** 2012-07-25

**Authors:** Janela McClure, Erica S. Lovelace, Shokrollah Elahi, Nicholas J. Maurice, Jessica Wagoner, Joan Dragavon, John E. Mittler, Zane Kraft, Leonidis Stamatatos, Helen Horton, Stephen C. De Rosa, Robert W. Coombs, Stephen J. Polyak

**Affiliations:** 1 Department of Laboratory Medicine, University of Washington, Seattle, Washington, United States of America; 2 Department of Medicine, University of Washington, Seattle, Washington, United States of America; 3 Department of Global Health, University of Washington, Seattle, Washington, United States of America; 4 Department of Microbiology, University of Washington, Seattle, Washington, United States of America; 5 Vaccine and Infectious Disease Division, Fred Hutchinson Cancer Research Center, Seattle, Washington, United States of America; 6 Viral Vaccines Program, Seattle Biomed, Seattle, Washington, United States of America; University of Pittsburgh Center for Vaccine Research, United States of America

## Abstract

Purified silymarin-derived natural products from the milk thistle plant (*Silybum marianum*) block hepatitis C virus (HCV) infection and inhibit T cell proliferation in vitro. An intravenous formulation of silibinin (SIL), a major component of silymarin, displays anti-HCV effects in humans and also inhibits T-cell proliferation in vitro. We show that SIL inhibited replication of HIV-1 in TZM-bl cells, PBMCs, and CEM cells in vitro. SIL suppression of HIV-1 coincided with dose-dependent reductions in actively proliferating CD19+, CD4+, and CD8+ cells, resulting in fewer CD4+ T cells expressing the HIV-1 co-receptors CXCR4 and CCR5. SIL inhibition of T-cell growth was not due to cytotoxicity measured by cell cycle arrest, apoptosis, or necrosis. SIL also blocked induction of the activation markers CD38, HLA-DR, Ki67, and CCR5 on CD4+ T cells. The data suggest that SIL attenuated cellular functions involved in T-cell activation, proliferation, and HIV-1 infection. Silymarin-derived compounds provide cytoprotection by suppressing virus infection, immune activation, and inflammation, and as such may be relevant for both HIV mono-infected and HIV/HCV co-infected subjects.

## Introduction

Human immunodeficiency virus-1 (HIV-1) infects approximately 40 million people worldwide. The majority (28 milllion) of infected individuals live in sub-Saharan Africa, and approximately 1 million infected people live in the USA. While antiretroviral therapy (ART) prolongs life in chronically infected subjects, therapy is life-long. Moreover, chronic HIV infection is often associated with generalized activation of the immune system [1], [2], characterized by increased frequencies of T cells expressing activation markers, expression of pro-inflammatory cytokines, continuous immune cell turnover, culminating in T and B lymphocyte exhaustion. Novel therapies that both suppress HIV-1 infection and attenuate immune activation might help to reduce the inflammatory pathogenesis of AIDS-defining and non-AIDS-defining diseases.

Approximately 30% of HIV-1 infected individuals in Europe and the USA are co-infected with hepatitis C virus (HCV) [3], totaling 4–5 million co-infected people [4]. Moreover, co-infection rates increase to 90% in HIV-1-positive intravenous drug user populations [5]. Co-infected persons have higher viral titers of HCV [6], and accelerated progression of liver disease including fibrosis, cirrhosis, and hepatocellular carcinoma compared to mono-infected persons [3], [7], [8], [9]. Although ART can effectively control HIV-1, co-infected patients are at increased risk of accelerated hepatic fibrosis [10]. As such, the development of drugs that are effective against both HCV and HIV infection would be advantageous.

Silymarin is an extract from the seeds of the milk thistle plant, *Silybum marianum*. Silymarin has been used since ancient times to promote liver health, is one of the top 10 most popular natural products consumed by western society, and is the most commonly consumed botanical medicine reported in patients with chronic hepatitis C [11], [12]. Silymarin extract contains silibinin, which is a mixture of the flavonolignans silybin A (SA) and silybin B (SB). Silibinin has antioxidant, immunomodulatory, antiproliferative, antifibrotic, and antiviral activities [13], [14], [15] in a wide range of tissues and organs [16]. Silymarin and silibinin both inhibit HCV infection in cell culture by variably blocking viral entry, viral fusion, viral RNA and protein synthesis, HCV N5SB RNA dependent RNA polymerase activity, and virus transmission [13], [14], [17], [18], [19], [20]. An intravenous formulation of silibinin, SIL, retains many of the same antiviral properties of the parent silibinin including inhibiting HCV fusion, replication, and production of infectious progeny virus [20]. In addition, patients treated intravenously with SIL displayed log-fold reductions in viral load when given to HCV mono-infected patients [21]. Moreover, SIL has recently been shown to inhibit HCV and, to a lesser extent, HIV-1, in an HCV/HIV co-infected patient [22].

Because SIL inhibits both HCV infection and T cell proliferation, we hypothesized that SIL inhibits HIV infection. We therefore examined the effects of SIL on in vitro HIV-1 replication in human peripheral blood mononuclear cells (PBMCs), a T-cell line (CEM cells), and Hela-derived TZM-bl cells. We found that SIL inhibited HIV-1 in all cell types tested. Suppression of HIV-1 infection was associated with toxicity-independent slowing of T cell proliferation. SIL also suppressed the induction of activation markers on CD4 T cells. These data suggest that SIL attenuates cellular functions required for activation, proliferation, and HIV infection.

## Methods

### SIL

SIL is a major component of silymarin, and consists of an equimolar mixture of silybin A and silybin B. To make SIL soluble in aqueous solution, silybin A and silybin B were converted into disodium disuccinyl silybin A and disodium disuccinyl silybin B. Each milligram of SIL contains 88% silibinin-succinate. SIL was kindly provided by Ralf Torsten-Paul (Rottapharm/Madaus, Lonza, Italy) and was solubilized in PBS.

### Cells

TZM-bl cells are a HeLa-derived cell line that expresses the HIV receptor CD4, and co-receptor CXCR4, and CCR5 [23]. TZM-bl cells were obtained through the NIH AIDS Reagent Program (ARP), and all TZM-bl based infections were in the presence of 10 mg/ml DEAE-dextran (Sigma, St. Louis) [24]. PBMC were originally isolated from leukapheresis units from ten healthy HIV-1 seronegative donors under written informed consent according to University of Washington Institutional Review Board (UW IRB) regulations. For the current studies, our work on these archived PBMC samples was deemed by the UW IRB to not meet the federal regulatory definition of “human subject research”, based on the Code of Federal Regulations (CFR), Title 45, Public Welfare Department Of Health And Human Services, Part 46, Protection Of Human Subjects (45 CFR 46.102(f)). Thus, the UW IRB waived formal review and approval of this study. PBMC cryopreserved in liquid nitrogen were thawed, stimulated with 1.5 µg/ml PHA in IMDM media supplemented with 10% FBS, and cultured for three days. The CEM-T4 cell line [25] was obtained through the ARP. CEM cells were cultured in IMDM media supplemented with 10% FBS and maintained in log phase by sub-culturing every 3–4 days.

### Viruses

HIV-1 LAI [26] and HIV-1 BAL [27] virus stocks were generated in PHA-stimulated PBMC cultures. LAI and BAL represent prototype clade B CXCR4 and CCR5 co-receptor-using virus isolates, respectively. For PBMC-based assays, the infectious titers of the virus stocks were determined by end-point dilution in PHA-stimulated PBMC [28]. The TCID (tissue culture infectious dose) was derived using the Reed-Meunch statistical method [29]. For pseudovirus assays, viral stocks were generated in 293T cells (obtained from ATCC) by co-transfecting the viral plasmid containing the *env* of interest and Q23 *env* [30], [31] DNA, a subtype A HIV-1 proviral clone with a deletion in *env*, creating viruses capable of only one round of infection. Transfections were performed using Fugene (Promega, Madison, WI) at a 20∶1 w/w backbone/*env* (i.e., Q23 *env* DNA with *env*, respectively) ratio to create pseudovirus, as previously described [32]. After 48 h, supernatants were clarified, concentrated using an Amicon Ultra centrifugal 100 kDa MWCO cellulose membrane filter device (Millipore), and infectious titers were determined using TZM-bl cells. The CCR5 co-receptor-using pseudoviruses tested included Q23 *env*(QA013.70I.*env*.M12; abbreviated D013M12)), which contains a subtype D *env* sequence, and Q23 *env*(Q769*env*.h5; abbreviated D769), which contains a subtype A *env* sequence [30], [33], [34]. The plasmids containing the HIV-1 *envs* and Q23 *env* were obtained from the ARP.

### Inhibition of HIV-1 Infection of TZM-bl Cells

To test the ability of SIL to inhibit viral infection in TZM-bl cells, serial two-fold dilutions of SIL, in triplicate, were co-incubated with HIV-1 viruses (LAI, BAL or pseudoviruses) for 1 hour at 37°C followed by the addition of TZM-bl cells for a final MOI of 0.05. Infections were incubated for 48 hours and cells were lysed, processed, and analyzed for luciferase activity using Galacto-Light Plus reagents (Applied Biosystems, Foster City, CA) on a TopCount NXT microplate luminometer, with a read-out of relative light units (RLU). All viral inhibition assays were performed with two independent preparations of viral stocks, and the percent inhibition was calculated as the percent reduction in RLUs of a virus incubated with a given concentration of SIL compared to the same virus incubated with growth medium alone. Percent inhibition was then plotted against the logarithm of the SIL concentration and a dose-response curve was fitted to calculate the IC_50_, the concentration of SIL required to inhibit infection by 50%, as previously described [35].

### Infection of PBMCs and CEM Cells

For HIV-1 replication studies, PBMC were thawed, stimulated with 1.5 µg/ml PHA in IMDM media supplemented with 10% FBS, and cultured for three days. PHA-blasts were washed and resuspended in IMDM media containing 10% FCS and 10 IU/ml recombinant interleukin 2 (IL-2; PeproTek, Rocky Hill, NJ). Cells were plated and concurrently exposed to SIL and virus. Residual virus inoculum was washed out after 24 hours and cells fed with fresh medium containing IL-2 and SIL. Since CEM cells do not require activation to support HIV infection, the cells were immediately infected with virus and exposed to SIL. Culture supernatants were monitored for virus production with HIV-1 p24 antigen capture assay. HIV-1 production by PBMCs and CEM cells was measured by determining the p24 levels in culture supernatants using an in-house double-antibody sandwich ELISA [36]. A standard curve was generated for each assay plate using a p24 standard obtained from the AIDS Vaccine Program at the National Cancer Institute (NCI)-Frederick Cancer Research and Development Center (Frederick, MD). Absorbance was measured at a wavelength of 450 nm.

### Cell Cycle Analysis

Untreated and PHA-blasted PBMC cultures were incubated with increasing concentrations of SIL. After treatments, cells were brought to a concentration of 1×10^6^ cells/mL and incubated for 2 *h* in 10 uM 5-ethynyl-2′-deoxyuridine (EdU; Invitrogen). Cells were distributed to 96-well round-bottomed plates in 1x PBS. Cells were washed, and stained with AViD dye (Invitrogen) plus fluorescently conjugated monoclonal antibodies to CD8-PE-Cy5, CD20-PE (BD), and CD4-PE-Cy7 (Beckman Coulter). Cells were fixed for 15 *min* in 1% paraformaldehyde (PFA) in PBS, permeabilized in 1x Click-iT saponin-based permeabilization and wash reagent (Invitrogen). Cells were stained for EdU content using a 250 µl volume of Click-iT detection cocktail with Alexa647-conjugated azide (Invitrogen) for 30 *min*. Cells were stained intracellularly using fluorescently conjugated CD3-ECD mAb (Beckman Coulter). After staining, cells were washed and resuspended in 198 µl PFA with 2 µl of 10.1 um AccuCount Rainbow Fluorescent Particles (Spherotech). Cells were analyzed on an LSRII flow cytometer (BD) and resultant data were analyzed using FlowJo software (FlowJo, Inc.) before being processed in JMP (SAS Institute, Inc.).

### Cell Cycle and Marker Expression Analysis

PHA-treated PBMCs were incubated with SIL and IL-2. After treatments, cells were brought to a concentration of 1×10^6^ cells/mL and incubated for 2 *h* in 10 uM EdU (Invitrogen). Cells were distributed to 96-well round-bottomed plates in 1x PBS. Cells were washed, stained with AViD dye (Invitrogen), and fluorescently conjugated mAbs to CD14-Alexa700, CCR5-APC-Cy7 (BD), and CXCR4-FITC (R&D). Cells were fixed, permeabilized, washed, and stained for EdU content as described in the previous section. Cells were stained intracellularly with fluorescently conjugated mAbs for CD3 (Beckman Coulter), CD4, CD8, and CD20 (BD). Cells were washed and resuspended in FxCycle-Violet (Invitrogen) in 1x Click-iT saponin-based permeabilization and wash reagent and incubated for 30 *min*. Five microlitres of 10.1 um AccuCount Rainbow Fluorescent Particles (Spherotech) were added to the cells using reverse pipetting. Cells were immediately analyzed using an LSRII flow cytometer (BD) and resultant data were analyzed using FlowJo software (FlowJo, Inc.) before being processed in JMP (SAS Institute, Inc.).

### CFSE Proliferation Assay

CD4+ T cell proliferation was determined with 5,6-carboxyfluorescein diacetate, succinimidyl ester (CFSE, Invitrogen) dilution. CFSE-labelled PBMCs were stimulated for 24 hr with PHA (2 µg/ml). Half of the medium was then replaced with fresh medium containing IL-2 (50 U/ml) and incubated in the presence of SIL for 4–5 days. This was followed by cell surface staining for CD3 and CD4 (BD). Cells were fixed in PBS with 2% PFA and acquired on LSRII flow cytometer (BD) and a minimum of 100,000 events collected.

### T Cell Activation Markers

PBMCs were activated with either SEB (0.5 µg/ml) or PHA (2 µg/ml) for 24 hours, and treated with or without SIL for 12 hours. Viability dye and antibodies directed against CD3, CD4, HLA-DR, CD38, Ki67, and CCR5 (BD) were then incubated with cells. Twenty-five min following staining, cells were washed and fixed in PFA, and acquired by flow cytometry using a LSRII flow cytometer (BD) and analyzed with FlowJo software v7.2.2 (Tree Star).

### Statistical Analyses

Logarithmic transformation followed by four-parameter logistic regression analysis was used for determining the IC_50_s of SIL against BAL and LAI in the TZM assays. Comparisons between experimental conditions were evaluated by Student's t-tests.

## Results

**Figure 1:**
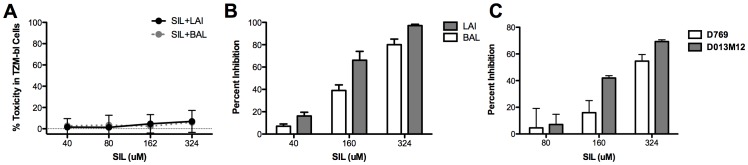
SIL suppresses HIV-1 Infection of TZM-bl cells. **A**, Cytotoxicity profile of SIL in TZM-bl cells. Cells were infected with LAI, a CXCR4-using virus, or BAL, a CCR5-using virus, at an MOI of 0.05 in the presence of the indicated concentrations of SIL and ATP was measured using the ATPlite kit 48 hours later. The data are representative of 2 (BAL) and 3 (LAI) independent technical repeats. **B**, Antiviral profile of SIL in TZM-bl cells. Serial dilutions of SIL were tested for inhibition of infection in TZM cells. Following addition of compounds and virus, cells were incubated for 48 hours before luciferase activity was measured. Percent inhibition refers to percent reduction in luciferase activity of SIL versus untreated cultures. Error bars represent standard deviation of 3 independent technical repeats. **C**, SIL inhibits pseudovirus replication in TZM-bl cells. TZM-bl cells were infected with the indicated viruses in the presence of the indicated concentrations of SIL and luciferase activity was measured 48 hours post-infection. The D013M12 psuedovirus contains a subtype D envelope sequence, while the D769 psuedovirus contains a subtype A envelope sequence. Error bars represent standard deviations of triplicate wells per condition.

We first examined the effects of SIL on HIV-1 replication in TZM-bl cells, which are a Hela-derived cell line that expresses the HIV receptor CD4, and co-receptor CXCR4, and CCR5 [23]. Since silymarin-derived compounds can induce apoptosis at high concentrations [37], we first performed SIL toxicity studies on TZM-bl cells. **Figure 1A** shows the effect of SIL, in the presence of BAL or LAI viruses, on ATP production, a sensitive marker of cell viability and growth [38] in TZM cells. SIL was non-toxic at all concentrations tested up to 324 µM.

**Figure 1B** shows that SIL suppressed infection of TZM-bl cells with LAI and BAL virus isolates. Inhibition was dose-dependent for both viruses, with a 97% suppression of LAI and 80% suppression of BAL and LAI at the highest dose of 324 µM. The average IC_50_ values for SIL inhibition of LAI and BAL were 146 µM and 266 µM, respectively (p<0.05). Thus, LAI appeared to be more susceptible than BAL to inhibition by SIL. SIL also suppressed the replication of HIV-1 pseudoviruses with clade A (the D769 virus) and clade D (the D013M12 virus) envelope sequences up to a maximum of 55% (for D769) and 70% (for D013M12) (**Figure 1C**), indicating that SIL inhibited different HIV clades. Since both BAL and the two tested pseudoviruses use CCR5 to enter cells, the data further suggest that CCR5-using viruses are less susceptible to inhibition by SIL than viruses that use CXCR4 for entry.

**Figure 2:**
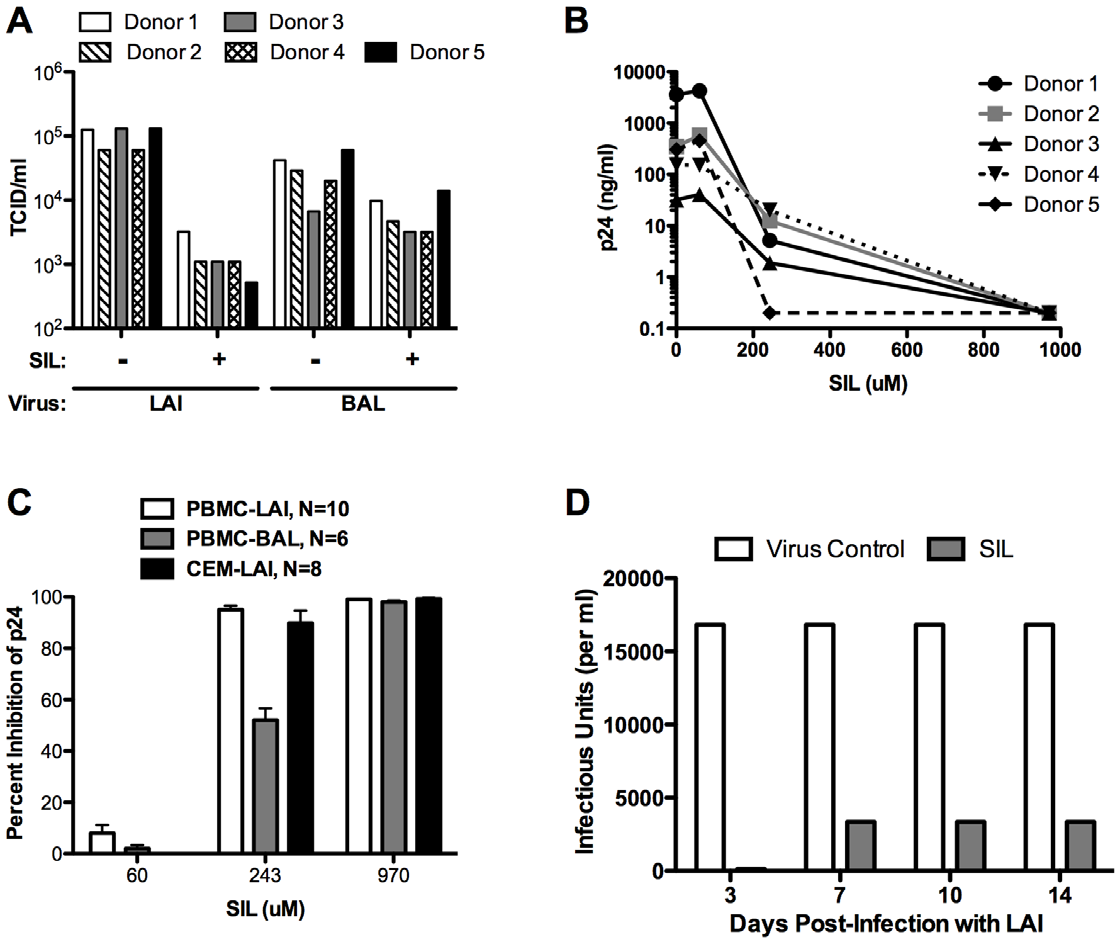
SIL inhibits HIV-1 infection in primary cells and a T cell line. **A**, SIL inhibits LAI and BAL infection of PBMCs derived from 5 different donors. PBMC were activated with PHA and 3 days later, cells were washed to remove PHA and resuspended in media containing IL-2. The cells were plated in 96 well plates in the presence or absence of 243 µM SIL. Cells were then infected with 3-fold serial dilutions of LAI and BAL virus stocks until the end point dilution was reached. Cultures were incubated 24 hours, input virus removed, and cultures were fed with media containing IL-2 and SIL. Supernatants were harvested 6 days later and assayed for p24 levels using HIV p24 Antigen Capture ELISA. **B**, Dose response of LAI inhibition by SIL. The five PBMC cultures were treated as described above and infected with LAI in the presence of the indicated concentrations of SIL. p24 ELISA was performed at 7 days post-infection. **C**, SIL inhibits HIV-1 infection of PBMCs and CEM cells. PBMCs were treated as described above. CEM cells were infected with LAI (MOI = 0.001) in the presence of the indicated amounts of SIL and p24 antigen was measured in culture supernatants at 4–7 days post-infection. The data represent pooled data from individual experiments of PBMCs infected with LAI (N = 10) or BAL (N = 6) and CEM infected with LAI (N = 8). **D**, SIL's anti-HIV effects are durable. PBMCs were activated and infected with LAI in the presence of 243 µM of SIL and p24 antigen was measured at the indicated times (days post-infection). Cells were fed every 3–4 days with medium containing fresh SIL. Virus control refers to cells that only received virus and no SIL.

We next examined the effects of SIL on HIV-1 replication in peripheral blood mononuclear cells (PBMC). **Figure 2** shows the inhibitory profile of SIL against BAL and LAI viruses in 5 different donor PBMC preparations. At a single dose of 243 µM, SIL inhibited both viruses although SIL appeared to consistently inhibit the LAI virus more effectively than the BAL virus (**Figure 2A**). SIL inhibited LAI in PBMCs in a dose-dependent fashion (**Figure 2B**), with doses of 243 µM and higher causing significant suppression relative to untreated cells (p<0.05). SIL also inhibited LAI infection of CEM cells in a dose dependent fashion (**Figure 2C**). These data were highly reproducible in PBMCs and CEM cells (**Figure 2C**). Moreover, SIL suppression of LAI infection in PBMCs was sustained for at least 2 weeks of culture (**Figure 2D**).

**Figure 3:**
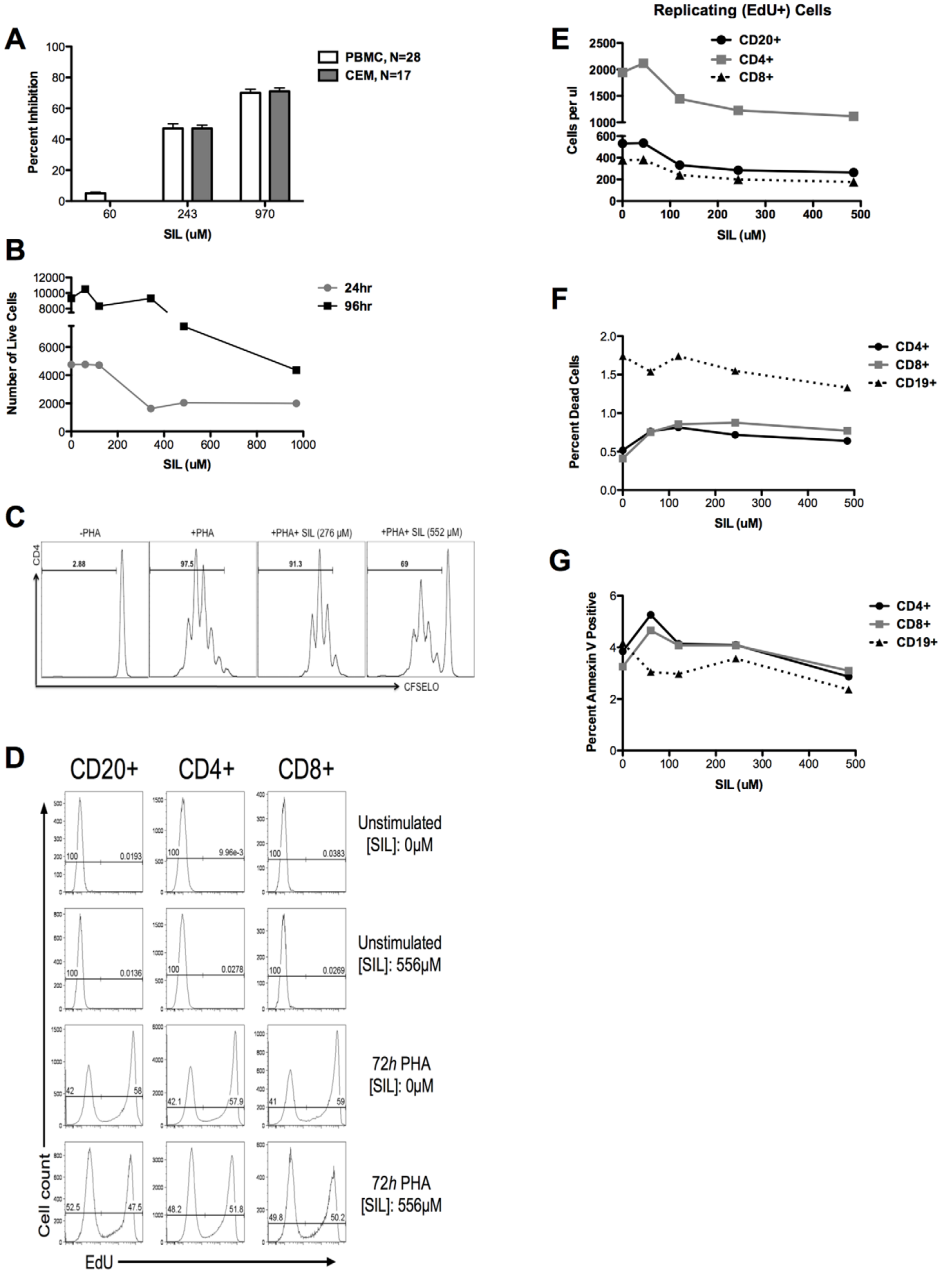
Suppression of HIV-1 by SIL correlates with inhibition of PBMC and CEM growth. **A**, SIL suppresses growth of PBMC and CEM cells. Cells were treated with SIL as described in the Materials and Methods and cells were counted 24 hours later by hemacytometer and trypan blue. **B**, Suppression of PHA-stimulated PBMC cell growth. After 24 hours of PHA stimulation, PBMC were placed in culture for 24 or 96 hours and the concentration of live cells per µL of sample was measured by flow cytometry. **C**, Suppression of CD4+ T cell proliferation measured by CFSE staining. PBMCs were labeled with CFSE and stimulated with PHA (2 µg/ml) for 24 hours prior to incubation with SIL for 4 days. **D**, SIL suppresses growth of T Cells and B Cells. Three day PHA-stimulated PBMC were treated with or without SIL for 24 hours and analyzed by flow cytometry following staining of T (CD4 or CD8) and B (CD20) cells and labeling of DNA using the Click-iT® EdU labeling kit. Peaks on the left of each graph represent EdU-, non-proliferating cells, while peaks on the right represent EdU+ proliferating cells. Top two rows depict unstimulated PBMC with and without SIL. Bottom two rows depict 72 *h* PHA and 24 *h* IL-2 stimulated PBMC with and without SIL. **E**, the number of EdU+ proliferating cells is inhibited by SIL. Cells were treated as described for panel C following exposure to SIL. The cell concentration is expressed per µl of cell suspension and is determined using counting beads as for panel B. **F, G**, SIL is not cytotoxic to PBMCs. PBMCs were stimulated with PHA for 3 days and treated with IL-2 in the presence of varying doses of SIL and 24 hours later, stained for CD4+, CD8+ and CD19+ (B cell) immune cell subsets and dead cells (**F**) as well as Annexin V+ apoptotic cells (**G**) were quantified.

Since we have previously shown that silymarin, silymarin-derived compounds, and SIL reduced T cell proliferation [13], [14], [17], [20], [39], we determined if SIL inhibition of HIV-1 infection correlated with reduced cellular proliferation. Indeed, SIL's anti-HIV-1 effects coincided with a reduction in absolute cell numbers of PBMCs and CEM cells, measured by hemocytometer counting (**Figure 3A**) or flow cytometric counting of PBMC numbers (**Figure 3B**), and by CSFE staining of CD4+ T cells (**Figure 3C**). Furthermore, SIL reduced cellular proliferation, measured as reduced EdU incorporation into DNA, in CD20+ B cells and CD4+ and CD8+ T cells (**Figure 3C** with quantification in **Figure 3D**). **Figure S1** depicts the gating tree for this experiment and verifies that all staining patterns were normal. Inhibition of IL-2-induced proliferation of CD20+, CD4+, and CD8+ cells was maximal with 243 µM SIL and plateaued with higher doses (**Figure 3E**). However, the reduction in PBMC proliferation observed after 24 hours exposure to SIL was not associated with increased cytotoxicity of CD20+, CD4+, or CD8+ cells in terms of necrotic cells (**Figure 3F**) or annexin V+ apoptotic cells (**Figure 3G**). Although there was a small increase in percent of dead cells and Annexin V+ at the lowest dose of SIL, the magnitude of this increase was likely not meaningful (from 0.5 to 0.8% for dead cells and 4 to 5% for Annexin V+ cells) and there was no trend toward more dead cells at higher SIL doses. Furthermore, DNA content analysis in CEM T cells after SIL exposure revealed no alterations in the percentages of cells in G1, S, or G2 phases of the cell cycle (**Figure S2**). The data indicate that SIL inhibited the growth of T and B cells but this was not due to toxicity via cell cycle arrest, apoptosis or necrosis.

**Figure 4:**
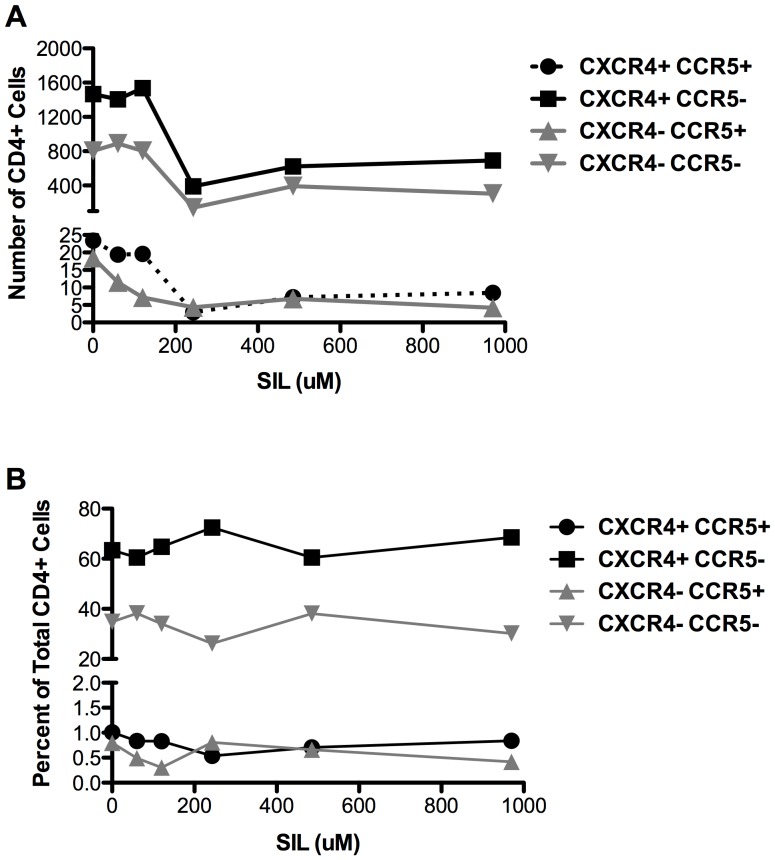
SIL inhibits stimulus-induced expansion of CD4+ T cells expressing the HIV co-receptors CXCR4 and CCR5 (panel A) but does not affect the relative frequency of CD4+ T cells expressing co-receptors (panel B). PBMCs were stimulated with PHA for 3 days prior to exposure to IL-2 and the indicated concentrations of SIL. Twenty-four hours later, cells were stained for CD4, CXCR4, and CCR5 and analyzed by flow cytometry. A, Y-axis represents the concentration of the indicated cell type. The cell concentration is expressed per µl of cell suspension and was determined using counting beads. Panel B shows the percentage of total CD4+ T cells that express one, both, or neither co-receptor.

Consistent with the observed decrease in absolute numbers of T cells, SIL reduced the number of CD4+ T cells expressing the HIV co-receptors CXCR4 and CCR5 (**Figure 4A**). The number of CD4+ and CD8+ (data not shown) T cells that were CXCR4+/CCR5+, CXCR4+/CCR5−, and CXCR4−/CCR5− was reduced, with maximal suppression by SIL at 243 µM, followed by a plateau at higher concentrations. However, the relative frequencies of CD4+ T cells expressing the various combinations of co-receptors were not affected by SIL (**Figure 4B**). The data indicate that SIL caused a generalized suppression of T cell expansion, resulting in fewer target cells with CD4 and CXCR4 and/or CCR5 that are susceptible to virus infection.

**Figure 5:**
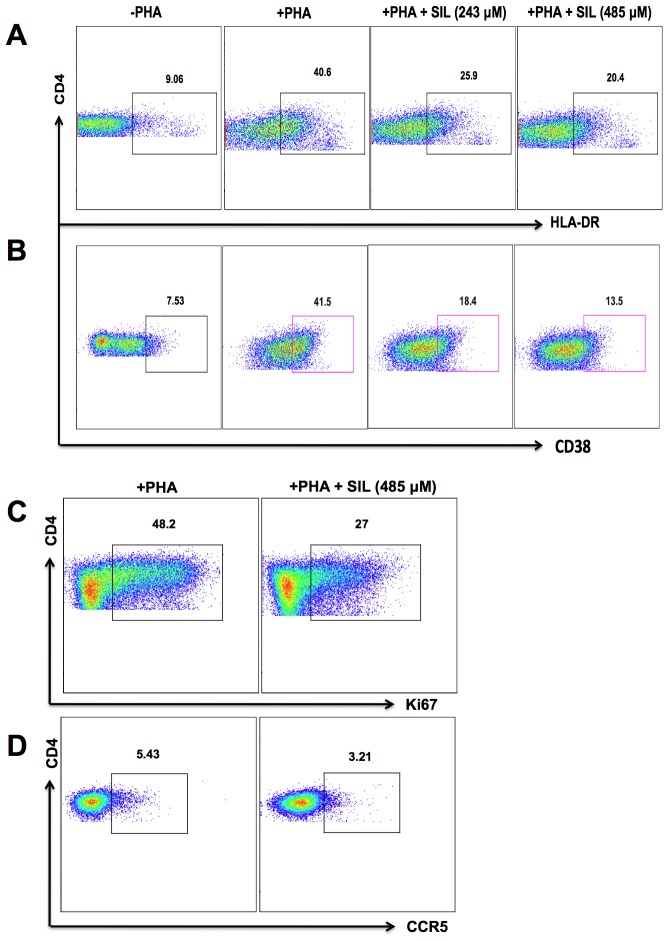
SIL inhibits activation marker expression on CD4+ T cells. PBMCs were activated with either SEB (0.5 µg/ml) or PHA (2 µg/ml) for 24 hours, and treated with the indicated concentrations of SIL for 12 hours. Representative flow cytometry dot plots showing expression of HLA-DR (A), CD38 (B), Ki67 (C), and CCR5 (D). Data are representative of 3 HIV-seronegative individuals tested for each marker.

To further explore the molecular mechanisms of how SIL attenuated T cell growth and HIV infection, we examined the effect of SIL on the expression levels of HIV-1 co-receptors and activation markers. Since CD4^+^ T cells from some donors expressed low levels of HIV co-receptors or activation markers, we induced expression of these molecules *ex vivo*. PBMCs were cultured in the presence of PHA or SEB and IL-2 for 24 hours to up-regulate CD38, HLA-DR, Ki67, and CCR5. Following activation, cells were cultured in the presence of SIL for 12 hours. As shown in **Figure 5**, SIL inhibited the expression of activation markers on CD4+ T cells including HLA-DR (Panel **A**), CD38 (Panel **B**), Ki67 (Panel **C**), and CCR5 (panel **D**), compared with untreated cultures. These data suggested that SIL's suppression of T cell proliferation and activation included reduced expression of T cell activation markers.

## Discussion

This study shows for the first time that a mixture of two compounds derived from silymarin and formulated for intravenous dosing inhibited HIV-1 infection in multiple cell types including human PBMCs, CEM cells, and TZM-bl cells. SIL's anti-HIV effects were seen against four viruses. The antiviral effect of SIL in T cells was associated with a reduction of stimulus-induced (PHA, IL-2) expansion of PBMC and primary CD4+ T cell cultures, and a reduction in CEM cell expansion in the absence of stimulation. Thus, SIL caused a generalized attenuation of T cell functions required for proliferation, activation potential and/or status, and HIV infection. The fact that SIL inhibited the expansion of both T and B cell subsets (Figure 3) and reduced the number but not proportions of CD4+ T cells expressing CXCR4 and/or CCR5, suggests that effects of SIL are generalized and not confined to a particular T cell subset. Therefore, as for HCV infection, SIL may modulate fundamental metabolic processes that result in the blunting of multiple cellular responses including activation and proliferation, which ultimately renders CD4^+^ T cells less susceptible to HIV-1 infection.

SIL suppression of T cell expansion did not involve cell death via cell cycle arrest, apoptosis or necrosis, nor inhibition of T cell subsets within PBMC cultures. This observation extends previous findings that in the context of HCV infection, silymarin, SIL, and silymarin-derived pure compounds inhibit T cell growth [13], [17], [20]. While SIL inhibited both primary and tumor T cells to similar degrees, SIL did not seem to inhibit the growth of adherent cells such as TZM-bl cells (as shown in this study) and human hepatoma Huh7 cells [20]. Additional studies are required to determine if SIL causes subtle growth and/or metabolic responses in adherent HIV-1 target cells, such as macrophages.

The current report focused on in vitro infection of PBMCs from HIV seronegative subjects. We speculate that since silymarin and silymarin-derived compounds and mixtures appears to block T cell activation by many stimuli including IL-2 (this study), PHA [17], and by anti-CD3 stimulation [13], [14], [20], we predict that SIL will also block stimulus-induced activation of virus replication in chronically infected cells.

Cumulatively, the data presented in this report suggest that SIL inhibited viruses by multiple inhibitory mechanisms that target the cell and reduce the activation status/potential. However, it is possible that SIL could hit virus-specific targets, because SIL and other silymarin-derived compounds are weak inhibitors of HCV NS5B polymerase activity in vitro [14], [18], [19], [20]. At present, we can only speculate which point(s) in the HIV lifecycle are impaired by SIL. This is particularly important because we showed that SIL inhibited a CXCR4 using virus (LAI) more effectively than CCR5-using viruses (BAL, and two pseudoviruses), yet SIL did not cause a preferential down-regulation of CXCR4 over CCR5 co-receptors. However, since we only tested a single CXCR4-using virus in the present study, some caution in interpreting these results is warranted. While SIL inhibits HCV entry by blocking fusion of viral membranes with endosomal membranes as well as transmission of progeny viruses [18], [20], HIV enters cells by fusion of viral and plasma membranes [40]. However, we have recently found that silibinin (the parent mixture of SIL) and SIL inhibits clathrin-mediated endocytosis and thereby inhibits viruses like HCV, vesicular stomatitis virus, reovirus, and influenza virus, which exploit the clathrin pathway to gain entry into cells (manuscript in preparation). Since HIV may also use clathrin to enter and traffic within cells [41], [42], SIL could inhibit HIV entry, possibly by blocking clathrin. Additional studies will be required to define where SIL acts on the HIV lifecycle and what accounts for the apparent differential suppression of CXCR4- versus CCR5-using viruses.

It has been previously shown that in both humans and in non-human primates, HIV-1 causes immune depletion at submucosal sites, such as the gastrointestinal track, followed by the onset of generalized immune activation [1], [2], manifested as increased frequencies of T cells expressing activation markers, increased expression of pro-inflammatory cytokines, continuous immune cell turnover, culminating in T and B lymphocyte exhaustion. Since SIL reduced expression of activation markers on CD4+ T cells (Figure 5), the immunomodulatory effects of SIL may be clinically relevant for reducing HIV-induced immune activation. In addition, because SIL blocks HCV re-infection during liver transplantation [43], [44] as well as HCV entry into hepatocytes [18], [20], silymarin-derived compounds and mixtures might have utility as a component of topical microbicide therapy [40] to prevent HIV infection, provided these compounds can be shown to inhibit HIV entry. Further investigation of the effects of silymarin-derived compounds in immune activation and transmission of HIV at mucosal sites is warranted.

In patients with HIV/HCV co-infections, T-cell activation can be higher than in patients infected with HIV alone [4]. Because silymarin and SIL suppress T cell proliferation [13], [14], [17], [20], silymarin-derived compounds may also be useful in controlling immune activation in co-infected patients. Moreover, silymarin-derived compounds and mixtures such as SIL may help reduce both HIV- and HCV-induced oxidative stress. For example, HCV infection induces oxidative stress and inflammation [45], and silymarin-derived compounds block HCV-induced oxidative stress directly by inhibiting virus replication as well as independently of suppression of viremia [14]. For HIV, there are several examples of gp120-mediated neurotoxicity involving oxidative stress, as well as numerous studies supporting the concept that oxidative stress is involved in the progression of HIV disease [46]. Therefore, silymarin-derived compounds may represent novel components of a multi-pronged approach to eradicate HCV, suppress HIV replication and transmission, and reduce immune activation and inflammatory disease in subjects with chronic HIV and/or HCV infection.

## Supporting Information

Figure S1 **Flow cytometry gating example for PHA-stimulated PBMC.** The two upper rows show the gating hierarchy to identify CD4+ and CD8+ T cells. The first gate excludes events during the first 10 to 15 seconds since pressure instability at the beginning of collection can affect fluoresence. The counting beads are visible as high for side scatter (SSC). Singlets are gated by forward scatter (FSC) area versus height. The AViD marker excludes dead cells. A large lymphocyte gate is used to capture blasting cells. Other lineage gates are as shown. The lower graphs show the expression of EdU and DAPI (DNA stain) for the three lineages. The lower right graphs show the expression of CXCR5 and CCR5.
(PDF)

Figure S2 **SIL does not induce cell cycle arrest.** CEM cells were incubated with the indicated concentrations of SIL and 72 hours later, cells were stained with DAPI and DNA content was analyzed by flow cytometry. Data represent the percent of cells in G1, S, and G2 phases of the cell cycle. This histograms represent raw data for cells treated without (left) and with 970 µM SIL (right).
(PDF)
