# Correction: Silibinin Inhibits HIV-1 Infection by Reducing Cellular Activation and Proliferation

**DOI:** 10.1371/annotation/78ba072a-6b7a-430a-8fcd-ef020e4fc458

**Published:** 2012-10-18

**Authors:** Janela McClure, Erica S. Lovelace, Shokrollah Elahi, Nicholas J. Maurice, Jessica Wagoner, Joan Dragavon, John E. Mittler, Zane Kraft, Leonidas Stamatatos, Helen Horton, Stephen C. De Rosa, Robert W. Coombs, Stephen J. Polyak

The ninth author's name was spelled incorrectly. The correct name is: Leonidas Stamatatos.

Additionally, the correct spelling of the name of the individual who provided SIL, mentioned in the SIL section of Methods, is Ralf Torsten-Pohl. 

