# Supplementary figures and images for: Silibinin Inhibits HIV-1 Infection by Reducing Cellular Activation and Proliferation
Source: PLoS One. 2012 Jul 25;7(7):e41832. doi: 10.1371/journal.pone.0041832 (PMC3404953; doi:10.1371/journal.pone.0041832)

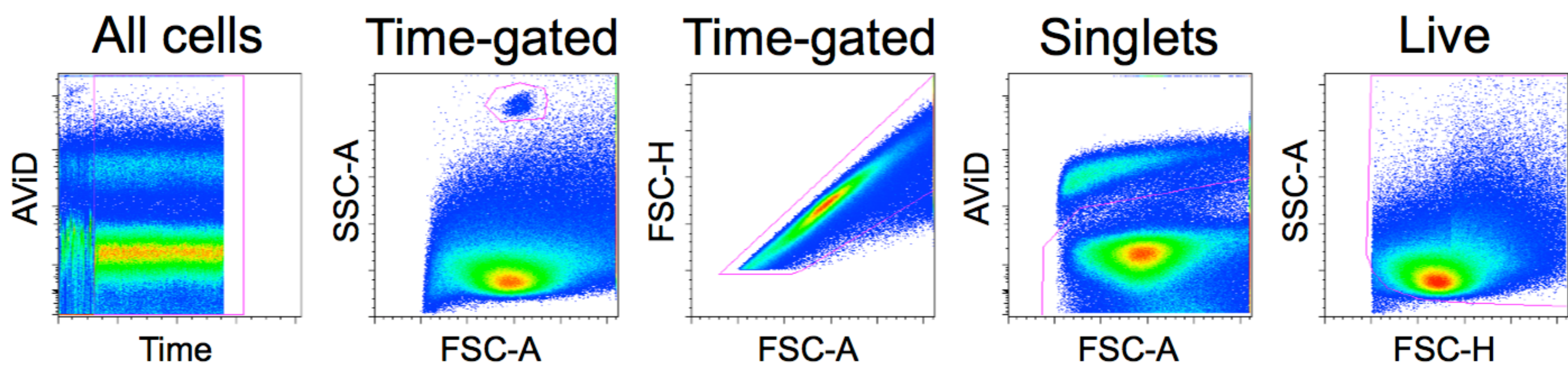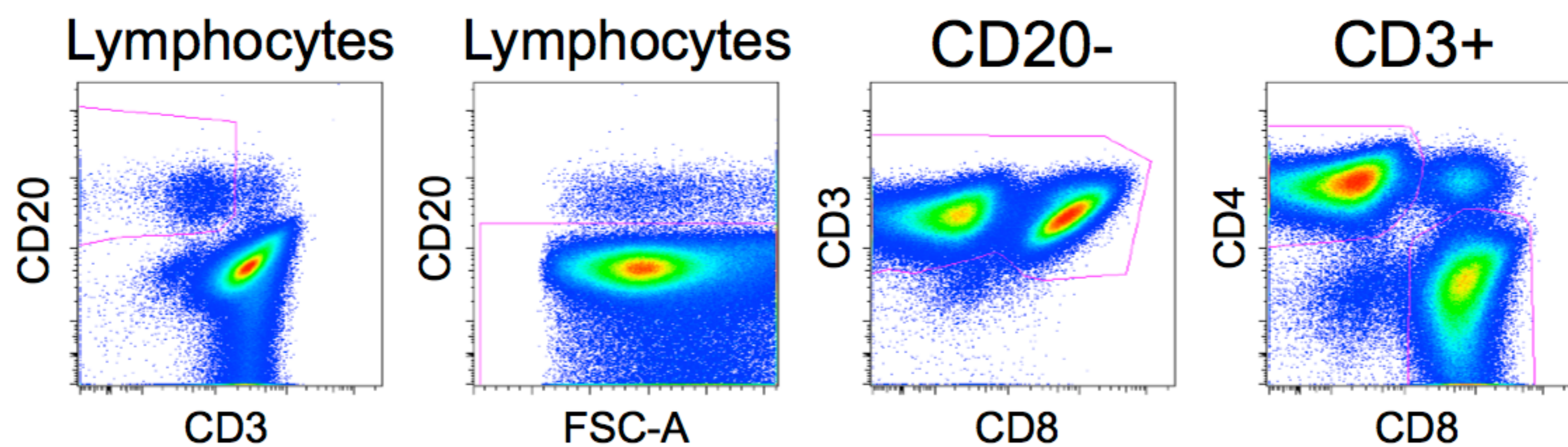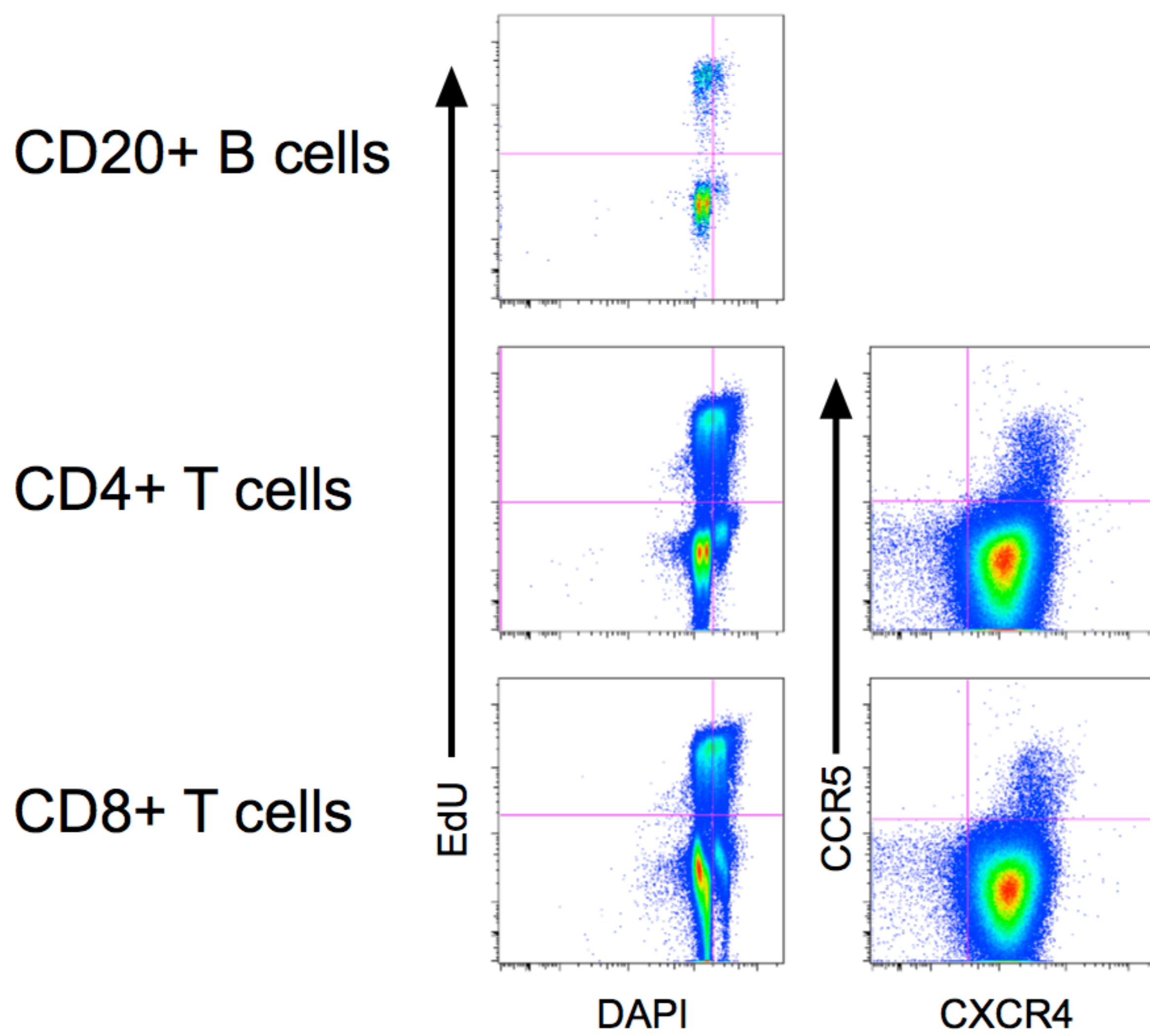

Supplement: Figure S1 — Flow cytometry gating example for PHA-stimulated PBMC. The two upper rows show the gating hierarchy to identify CD4+ and CD8+ T cells. The first gate excludes events during the first 10 to 15 seconds since pressure instability at the beginning of collection can affect fluoresence. The counting beads are visible as high for side scatter (SSC). Singlets are gated by forward scatter (FSC) area versus height. The AViD marker excludes dead cells. A large lymphocyte gate is used to capture blasting cells. Other lineage gates are as shown. The lower graphs show the expression of EdU and DAPI (DNA stain) for the three lineages. The lower right graphs show the expression of CXCR5 and CCR5. (PDF) [file pone.0041832.s001.pdf]

72 hours

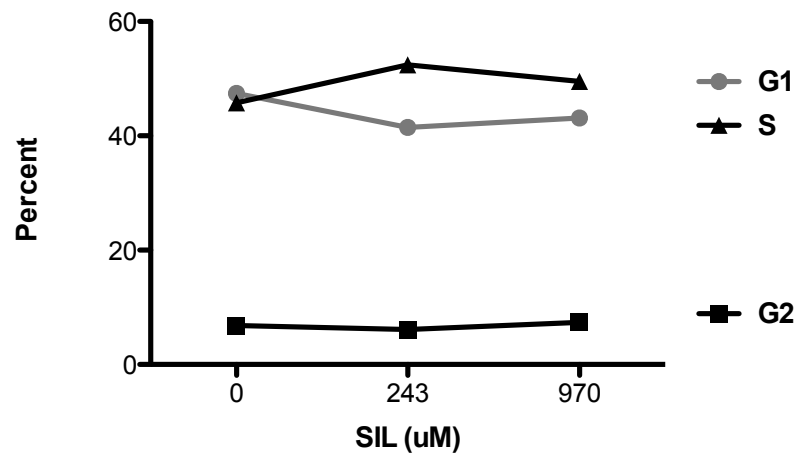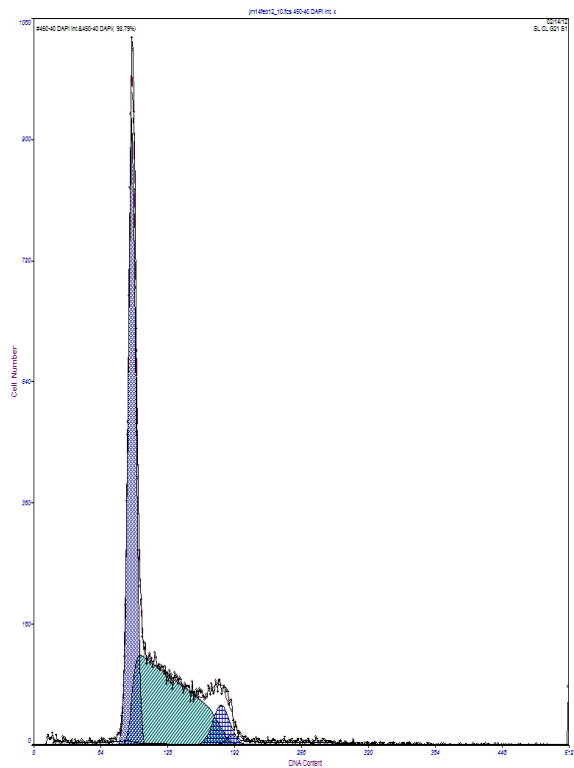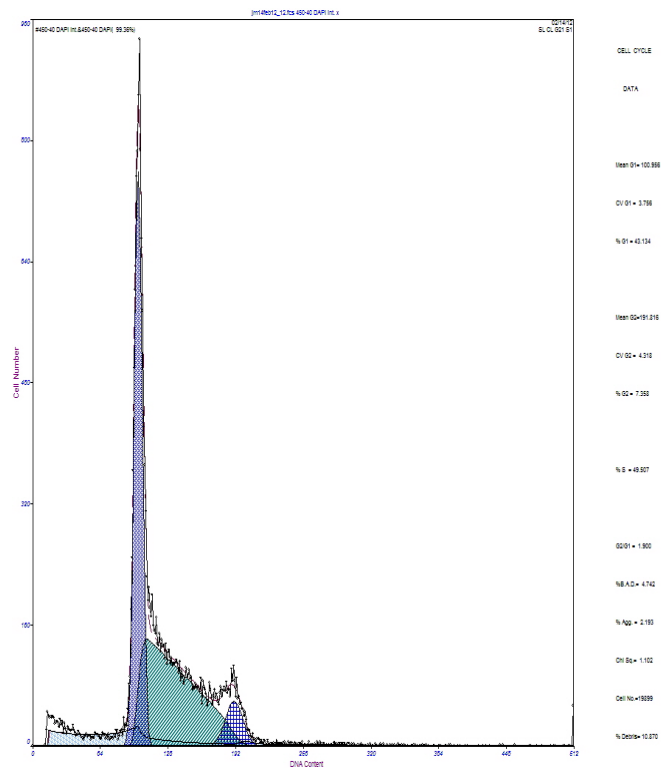

Supplement: Figure S2 — SIL does not induce cell cycle arrest. CEM cells were incubated with the indicated concentrations of SIL and 72 hours later, cells were stained with DAPI and DNA content was analyzed by flow cytometry. Data represent the percent of cells in G1, S, and G2 phases of the cell cycle. This histograms represent raw data for cells treated without (left) and with 970 µM SIL (right). (PDF) [file pone.0041832.s002.pdf]
